# Respiratory-related death in individuals with incident asthma and COPD: a competing risk analysis

**DOI:** 10.1186/s12890-022-01823-4

**Published:** 2022-01-08

**Authors:** Alicia V. Gayle, Cosetta Minelli, Jennifer K. Quint

**Affiliations:** 1 National Heart and Lung Institute, Imperial College London, G08 Emmanuel Kaye Building, Manresa Road, London, SW3 6LR UK; 2 Imperial Biomedical Research Center, National Institute for Health Research, London, UK; 3 AstraZeneca PLC, Cambridge, UK

**Keywords:** COPD, Asthma, ACO, Mortality

## Abstract

**Background:**

Distinguishing between mortality attributed to respiratory causes and other causes among people with asthma, COPD, and asthma-COPD overlap (ACO) is important. This study used electronic health records in England to estimate excess risk of death from respiratory-related causes after accounting for other causes of death.

**Methods:**

We used linked Clinical Practice Research Datalink (CPRD) primary care and Office for National Statistics mortality data to identify adults with asthma and COPD from 2005 to 2015. Causes of death were ascertained using death certificates. Hazard ratios (HR) and excess risk of death were estimated using Fine-Gray competing risk models and adjusting for age, sex, smoking status, body mass index and socioeconomic status.

**Results:**

65,021 people with asthma and 45,649 with COPD in the CPRD dataset were frequency matched 5:1 with people without the disease on age, sex and general practice. Only 14 in 100,000 people with asthma are predicted to experience a respiratory-related death up to 10 years post-diagnosis, whereas in COPD this is 98 in 100,000. Asthma is associated with an 0.01% excess incidence of respiratory related mortality whereas COPD is associated with an 0.07% excess. Among people with asthma-COPD overlap (N = 22,145) we observed an increased risk of respiratory-related death compared to those with asthma alone (HR = 1.30; 95% CI 1.21–1.40) but not COPD alone (HR = 0.89; 95% CI 0.83–0.94).

**Conclusions:**

Asthma and COPD are associated with an increased risk of respiratory-related death after accounting for other causes; however, diagnosis of COPD carries a much higher probability. ACO is associated with a lower risk compared to COPD alone but higher risk compared to asthma alone.

**Supplementary Information:**

The online version contains supplementary material available at 10.1186/s12890-022-01823-4.

## Background

Hundreds of millions of people worldwide are diagnosed with chronic respiratory diseases, and approximately four million premature deaths are attributed to these conditions each year [1]. Asthma and chronic obstructive pulmonary disease (COPD) are the most prevalent chronic respiratory diseases, affecting 358 and 174 million people respectively. Approximately 180,000 deaths worldwide each year are attributable to asthma [2], a figure that has fallen substantially in recent decades with the introduction of guidelines for treatment that emphasise the use of inhaled steroids to control the disease [3]. Deaths from COPD however, are eight times more common than deaths from asthma [4], and are considered the fourth leading cause of death worldwide [1]. Around 11% of people have overlapping diagnoses of asthma and COPD (ACO), and people with ACO may have worse outcomes than those with either asthma or COPD alone [5–8]. Globally, rates of respiratory-related death have decreased over time [9, 10]. However, compared to other European countries, the UK has consistently had the highest age-standardised mortality rates attributed to respiratory disease [11].

Our previous research has shown that respiratory-related deaths have remained constant over time among people with asthma and COPD while cardiovascular-related (CVD) deaths declined and deaths attributed to mental and behavioural disorders increased [12]. While these underlying shifts may be attributed to improved management in comorbid conditions, there is a need to estimate the risk of respiratory-related mortality after taking into account risk of death attributed to other causes; this would help to disentangle the risk of respiratory-related mortality from other competing causes of death and allow more accurate estimation of the mortality burden of people with asthma and COPD.

The aim of this study was to estimate the risk of respiratory-related death among those with a physician diagnosis of asthma or COPD compared to a population without asthma or COPD, using competing risk regression to account for the presence of “competing” causes of deaths. Furthermore, considering the paucity of epidemiological evidence around co-occurring asthma and COPD (ACO), we also estimated the risk of both overall and respiratory-related mortality of people with ACO compared to those with asthma or COPD alone.

## Methods

### Design

This matched cohort study analysed electronic healthcare data obtained from the UK Clinical Practice Research Datalink (CPRD) GOLD data (October 2017 build, see Additional file 1 for further details). Linked pseudonymised mortality data from the Office for National Statistics (ONS), and socioeconomic data from the Index of Multiple Deprivation (IMD) were provided for this study by CPRD for patients in England (linkage set 15).

We compiled a cohort of adults (aged ≥ 18) with an incident asthma or COPD physician diagnosis using pre-specified validated Read codes (S1, S2), who were registered to a GP practice contributing data between 2005 and 2015 and then included two groups of people from the total CPRD population who did not ever have a diagnosis of asthma or COPD as matched comparators, hereafter referred to as “the control groups”. People in control groups were only included if they were registered to practices that had consented to linkage with ONS data (the same criteria as for the COPD and asthma populations in the study). Individuals were frequency matched on age (5-year age band), sex and GP practice at a ratio of 5:1, with age considered at the asthma/COPD diagnosis date or index date for those in the control groups and exact matches prioritized.

An additional cohort of individuals with ACO was compiled. People with an asthma diagnosis followed by a COPD diagnosis (asthma at least 2 years prior to COPD, to avoid misdiagnosis [7]) (see Additional file 1), and people with a COPD diagnosis followed by an asthma diagnosis (COPD diagnosis at any time before asthma diagnosis). People with asthma or COPD were compared with the control groups, while people with ACO were compared to those with asthma alone and COPD alone. Follow-up time was determined from the date of diagnosis among people with asthma or COPD, and from the index date among people from the control groups. The end of follow-up was defined as the earliest of: the date of transfer out of the GP practice, the last date of data collection from that practice or death (S3).

Age was measured in years. Patient socioeconomic score was determined using linked CPRD-IMD 2015 data divided into quintiles, where 1 is most deprived and 5 is least deprived [13]. Baseline body mass index (BMI) was calculated using height and weight data from CPRD, where < 18.5 kg/m^2^ was ‘underweight’, 18.5–24.9 kg/m^2^ ‘healthy weight’, 25.0–29.9 kg/m^2^ ‘overweight’, and > 30.0 kg/m^2^ ‘obese’. Smoking status was classified, based on recorded status at the start of follow-up, as ‘never smoker’, ‘current smoker’, ‘former smoker’, and ‘Not recorded’. For all variables, missing information was analysed as a separate category, ‘Missing’. A total Charlson Comorbidity Index score [14] was calculated by summing comorbidities up to the time of diagnosis (index), scores were then grouped into categories [0, 1, 2, 3, 4,  ≥ 5].

### Outcome assessment

Mortality and the leading cause of death during follow-up were ascertained using patient underlying cause of death identified from the death certificates from the national mortality offices. The identification of the underlying cause of death is based on ICD rules and is made from the condition(s) reported by the certifier, as recorded on the certificate [15]. The underlying cause of death, as defined by WHO, is either the disease or injury that initiated the train of events directly leading to death, or the circumstances of the accident or violence that produced the fatal injury [16]. All deaths were grouped according to the ICD-10 chapter codes. Mortality was classified as being due to respiratory disease (ICD J00–J99), cardiovascular disease (ICD F01, G45, I00–I99, Q20, Q28, and R96), any malignant neoplasm (ICD C00–C99 and D1–D48), diseases of the digestive system (ICD K00–K93), mental and behavioural disease (ICD F00–F99), and other causes (ICD chapters A, D, E,G,H, L, M, O, P, N, Q, R,U, X).

### Statistical analysis

Baseline characteristics for the different disease groups were considered at the diagnosis/index date. Standardised differences in covariates between patients with and without disease were calculated to assess the balance of baseline characteristics obtained with the matching; standardised differences of < 0.1 suggests that these covariates are well balanced [17].

We used a competing risks analysis, in which we consider respiratory-related death associated with the respiratory disease of interest while accounting for other competing causes of death [18]. The subdistribution hazard ratios estimate the relative change in the instantaneous rate of respiratory-related death in those people who have not died or who have died due to a competing cause [19], and it is interpreted as the magnitude of the relative change in rate of respiratory-related death associated with the presence of the respiratory disease. This relies on the assumption that time to respiratory-related death is independent between people and on the assumption of proportionality between groups, (i.e. the difference in rate of respiratory-related death between those with disease and without disease remains constant over time). We assessed the proportional hazard assumption by plotting the Schoenfeld residuals and checking that the pattern against time was constant [20].

For each disease group, we estimated cause-specific mortality rates, expressed per 100,000 person-years. We then estimated the subdistribution hazard ratio (HR) for death and 95% confidence interval (95% CI) associated with asthma or COPD using Fine-Gray regression, implemented with the “stcrreg” command in STATA [21]. The regression models were adjusted for age, sex, smoking status, Body Mass Index (BMI), and socioeconomic status, as measured by the Index of Multiple Deprivation (IMD), comorbidities using the Charlson Comorbidity Index, and they accounted for deaths from other causes as competing risks.

We determined the cumulative incidence function (i.e., the predicted cumulative risk of death) by 10 years from diagnosis for each specific cause of death. The 10-year cut-off point was used, as the majority of deaths had occurred by then. We calculated the excess risk of death for each cause of death as the difference between the cumulative incidence of death for those with disease compared with those without disease.

Sensitivity analysis included estimation of respiratory mortality associated with overlapping asthma and COPD by order of diagnosis (S3). We compared overlapping groups to those who had asthma only and COPD only.

## Results

**Fig. 1:**
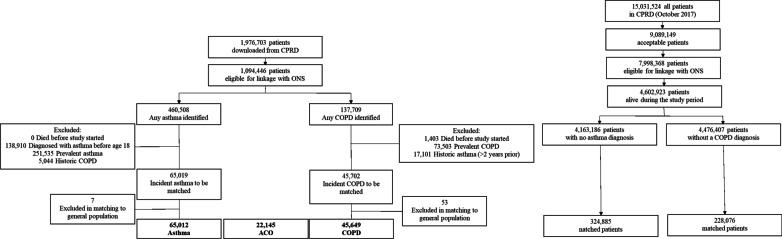
Flow Chart of patient inclusion. Office for National Statistics (ONS), asthma-COPD overlap (ACO), chronic obstructive pulmonary disease (COPD)

We initially identified 65,019 people with incident asthma, 45,702 with COPD. After frequency matching to the control groups, our final cohorts included 65,012 people with asthma, 45,649 COPD, and 22,145 with ACO (Fig. 1) (S4). The median follow-up was 3.0 years (Interquartile range (IQR): 2.3–5.6) and 4.0 years (IQR: 1.9–6.9) among people with asthma and without, and 3.8 years (IQR: 1.8–6.4) and 3.9 (IQR: 1.8–6.5) years for those with and without COPD, respectively (Table 1).

**Table 1:** Baseline characteristics

Characteristic	Asthma		Std. Diff	COPD		Std. Diff	ACO
	Yes	No		Yes	No		
	(N = 65,012)	(N = 324,885)		(N = 45,649)	(N = 228,076)		(N = 22,145)
*Sex (N, %)*							
Male	25,977 (40)	129,793 (40)	0.00	25,377 (56)	126,775 (56)	0.00	10,205 (46)
Female	39,035 (60)	195,092 (60)		20,272 (44)	101,301 (44)		11,940 (54)
Age at inclusion/diagnosis (mean, SD)	50.4 ± 18.2	50.4 ± 18.2	0.00	67.8 ± 11.5	67.8 ± 11.4	0.00	65.5 ± 12.1
*Age at inclusion/diagnosis (N, %)*							
18–44	26,434 (41)	132,105 (41)	0.00	1262 (3)	6307 (3)	0.00	1126 (5)
45–64	22,411 (35)	112,044 (35)		16,207 (36)	81,036 (36)		8890 (40)
65–84	14,481 (22)	72,385 (22)		25,185 (55)	125,911 (55)		11,003 (50)
> 85	1686 (3)	8351 (3)		2995 (7)	14,822 (7)		1126 (5)
Follow up time, years (Median (IQR))	3.0 (2.3–5.6)	4.0 (1.9–6.9)		3.8 (1.8–6.4)	3.9 (1.8–6.5)		3.8 (2.1–6.3)
Total person years	259,700	1,460,056		193,309	994,000		96,038
*Smoking status (N, %)*							
Never smoker	25,803 (40)	160,793 (50)	0.27	3,859 (9)	101,784 (45)	0.99	4373 (20)
Current	12,469 (19)	66,252 (20)		18,298 (40)	31,493 (14)		6912 (31)
Former smoker	22,374 (34)	88,313 (27)		21,560 (47)	90,698 (40)		10,860 (49)
Not recorded	4366 (7)	9527 (3)		1932 (4)	4101 (2)		0 (0)
*BMI (kg/m*^*2*^*)*							
Underweight (< 18.5)	1167 (2)	7406 (2)	0.22	2023 (4)	3084 (1)	0.22	552 (2)
Normal weight (18.5–24.9)	17,747 (27)	109,319 (34)		14,393 (32)	65,811 (29)		5996 (27)
Overweight (25–29.9)	20,119 (31)	97,373 (30)		14,030 (31)	84,417 (37)		7133 (32)
Obese (30 +)	19,736 (30)	71,499 (22)		12,264 (27)	57,943 (25)		2158 (10)
Missing	6243 (10)	39,288 (12)		2939 (6)	16,821 (7)		0 (0)
*IMD (N, %)*							
1 (least deprived)	13,583 (21)	73,699 (23)	0.06	7125 (16)	48,593 (21)	0.22	3565 (16)
2	13,837 (21)	70,574 (22)		8765 (19)	50,683 (22)		4325 (20)
3	13,224 (20)	67,034 (21)		9181 (20)	47,146 (21)		4450 (20)
4	12,550 (19)	60,676 (19)		10,006 (22)	42,979 (19)		4733 (21)
5 (most deprived)	11,765 (18)	52,574 (16)		10,524 (23)	38,476 (17)		5059 (23)
Missing	53 (0)	328 (0)		48 (0)	199 (0)		0 (0)
*CCI (N, %)*							
0	0 (0)	227,852 (70)	2.29	0 (0)	117,111 (51)	1.51	0 (0)
1	50,226 (77)	51,073 (16)		23,765 (52)	41,445 (18)		12,349 (56)
2	5637(9)	24,022 (7)		7457 (16)	31,724 (14)		3581 (16)
3	4712 (7)	11,531 (4)		6367 (14)	18,351 (8)		2954 (13)
4	2267 (4)	4993 (2)		3688 (8)	8912 (4)		1594 (7)
≥ 5	2170 (3)	5414 (2)		4372 (10)	10,533 (5)		1667 (8)

ACO: concomitant asthma and COPD, Std. Diff: Standardised Difference, SD: Standard Deviation, IQR: Interquartile Range, BMI: Body Mass Index, IMD: Index of Multiple Deprivation, CCI: Charlson Comorbidity Index

Compared to the control groups, people with asthma were more likely to be overweight or obese (61% among asthma vs 52% in the control groups), while those with COPD were more likely to smoke (40% vs 14% in the control groups) and to live in areas of greater deprivation (45% among COPD vs 36% in the control groups). Among people with overlap of the two conditions, individuals with ACO had a higher proportion of current smokers (31%) compared with individuals with asthma alone (19%) but not when compared to COPD alone (40%) (Table 1).

### Mortality rates

We observed 23,471 deaths during the follow-up period among individuals with a diagnosis of COPD or asthma and 75,739 among the control groups; the overall mortality rate was 2954 per 100,000 person-years among people with asthma and 8173 per 100,000 person-years among people with COPD (S4). Among those with asthma and COPD, the leading causes of respiratory death were COPD (53% among asthma patients, 63% among COPD patients) and pneumonia (16% among asthma patients, 11% among COPD patients) (S5). Mortality rates among those with overlapping diagnoses was 4485 per 100,000 person-years (S6). The overall mortality rate among people with asthma showed only a slight increase in overall mortality (2954 vs 2034 per 100,000 person-years) compared with the control group. This was significantly higher in individuals with COPD compared to the control group (8173 vs 4631 per 100,000 person-years) (S4). Mortality rates attributed to non-respiratory causes of death were consistent with previous findings [12].

**Fig. 2:**
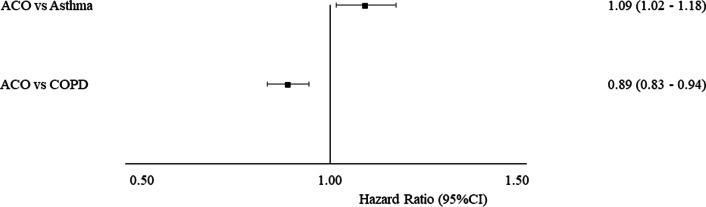
Subhazard of respiratory-related mortality between disease groups. asthma-COPD overlap (ACO), confidence interval (CI)

After adjusting for competing risks, compared to the control group we observed a higher risk of respiratory-related mortality for people with asthma (HR = 2.22, 95% CI 2.08–2.36), and people with COPD (HR = 3.31, 95% CI 3.15–3.47) (S5). Figure 2 shows the risk of respiratory-related death among those with a single disease diagnosis compared to those with overlap. Co-existence of asthma and COPD is associated with an increased risk of respiratory-related death when compared to asthma alone (HR = 1.30, 95% CI 1.21–1.40). However, when compared to COPD alone we saw a decreased risk (HR = 0.89 95% CI 0.83–0.94). When considering the order of overlapping diagnosis, having COPD followed by asthma was associated with a lower risk of respiratory-related death when compared to asthma and COPD (HR = 0.82, 95% CI 0.69–0.96 compared to asthma, HR = 0.67 95% CI 0.58–0.79 compared to COPD, S7).

### Cumulative incidence of death

At 10 years post diagnosis, only 14 in 100,000 asthmatics are predicted to experience a respiratory-related death, an excess of 9 in 100,000 (95% CI 0–18 in 100,000) compared to the control group (5 in 100,000). This was lower than the predicted incidence of deaths related to the circulatory system and neoplasms (18 and 43 in 100,000 respectively). A higher proportion of people with COPD are predicted to experience a respiratory related death (98 in 100,000 people) at 10 years post diagnosis, and this is higher than estimates observed among the control group (excess 67 per 100,000; 95% CI 38–95 in 100,000). This estimate was also lower than the predicted incidence of deaths related to the circulatory system and neoplasms (106 and 149 in 100,000 respectively) (Table 2).

**Table 2:** Cumulative incidence over 10 years (%) and excess cause-specific death adjusted for age, sex, BMI, smoking status, IMD and competing risks, by underlying respiratory disease

Cause of death	Cumulative incidence at 10 years		Excess (95% CI)	Cumulative incidence at 10 years		Excess (95% CI)
	Asthma (%)	Control group (%)		COPD (%)	Control group (%)	
Diseases of the respiratory system (J00–J99)	0.014	0.005	0.009% (− 0.000, 0.018)	0.098	0.031	0.067% (0.038, 0.095)
Diseases of the circulatory system (I00–I99)	0.018	0.014	0.003% (− 0.007, 0.013)	0.106	0.088	0.018% (− 0.012, 0.047)
Neoplasms (C00–D48)	0.043	0.034	0.009% (− 0.007, 0.025)	0.149	0.113	0.036% (0.001, 0.071)
Diseases of the digestive system (K00–K93)	0.007	0.005	0.002% (− 0.004, 0.008)	0.018	0.016	0.003% (− 0.010, 0.015)
Mental and behavioural disorders (F00–F99)	0.001	0.001	0.000% (− 0.003, 0.002)	0.008	0.016	− 0.008% (− 0.017, 0.000)
Other*	0.014	0.013	0.001% (− 0.008, 0.010)	0.039	0.041	− 0.002% (− 0.020, 0.016)

CI: confidence interval. COPD: Chronic Obstructive Pulmonary Disease

*Includes missing cause of death. Asthma (N = 65,012) compared to the control group (N = 324,885). COPD (N = 45,649) compared to the control group (N = 228,076)

## Discussion

People with incident physician-diagnosed adult-onset asthma have an increased risk of respiratory-related death compared to those without, but the probability of a respiratory-related death is much lower than among people with COPD. A physician diagnosis of COPD is strongly associated with an increased risk of respiratory-related mortality after controlling for competing risks. The most probable cause of death at 10 years after COPD diagnosis was respiratory disease when compared to the control group. Co-occurring asthma and COPD is associated with an increased respiratory-related mortality risk compared to asthma, but not when compared to COPD.

Our results confirm a low asthma mortality burden in the UK. Shaw et al. consider the absolute number of asthma and COPD deaths in England over the last 10 years and report a 53% reduction in the number of asthma deaths among people aged below 75, however these estimates are not age-standardised and use population mortality statistics alone [22]. Similarly, the National Review of Asthma Deaths (NRAD report) [13] highlighted that only a small proportion of the asthma population have an asthma-related death. Our study, based on a cohort of people with newly diagnosed adult-onset asthma, shows that while people with asthma are at an increased risk of respiratory-related mortality compared with those without asthma, the probability of a respiratory-related death at 10 years post diagnosis is relatively low, particularly when compared to the predicted incidence among those with COPD. This may be attributed to more effective treatment for asthma compared to COPD and also the differing natural history of both diseases. However, our study focuses on the incident adult diagnosed population and therefore results may not extend to those with childhood-onset disease. A longer period of follow up would bring the lifelong risk of respiratory-related death among asthmatics in line with that expected in those without asthma as the majority of people with asthma have mild disease, severe/uncontrolled asthma represents only 5–10% of the asthma population [24]. Use of inhaled corticosteroids rather than short-acting therapies alone, increased disease awareness and early intervention, would help to reduce the elevated risk [25].

Among people with COPD, our results are consistent with previous literature [26, 27]. Using a similar analytical approach, Abukhalaf et al. found that disease severity and smoking status were linked to risk of respiratory-related mortality when applying a competing risk approach to a cohort of 512 people in the US with COPD [26]. People with COPD are usually current or former smokers [28], older, and have comorbid conditions; whereas people with asthma are younger on average, less likely to smoke and so have an attenuated risk [29]. Furthermore, there are increased opportunities to misdiagnose COPD for example as heart failure, as both conditions are systemic disorders with overlapping pathophysiological processes [30, 31], and coexistence of both diseases has been shown to increase mortality risk [32]. This study provides further evidence that COPD is associated with a high respiratory-related mortality burden despite the widespread availability and routine use of disease management interventions such as pulmonary rehabilitation, smoking cessation programmes, use of prognostic risk calculators [33] partnered with targeted therapeutics.

Our results suggest that overlapping diagnoses of asthma and COPD is associated with an increased respiratory-related mortality risk compared to asthma, but not when compared to COPD. The latter was an unexpected finding as previous studies have found a consistently higher risk compared to single disease. In the National Health and Nutrition Examination Survey III (NHANES-III) cohort, Diaz-Guzman et al. [34] found that the hazard ratio for mortality in ACO was 1.8, compared with 1.4 in COPD and 1.2 in asthma, with COPD defined by spirometry. Similarly, Huang et al. found an increased mortality risk among those with airflow limitation and physician confirmed asthma compared to those without disease and when compared to those with COPD alone (FEV/FVC < 70%) [35]. However, in a cohort of spirometry confirmed ACO and COPD patients, Bai et al. concluded that patients with ACO were more likely to have better prognosis and lower mortality than those with COPD alone [36]. Similarly, Fu et al. observed poorer prognosis for patients with COPD compared to ACO [37]. The conflicting literature may be partly due to misclassification, or driven by the proportion of current smokers in each group [29]. Currently, clinical guidance considers asthma and COPD as different disorders despite common traits and clinical features (e.g., eosinophilia, some degree of reversibility)[37]. Our results show that risk of respiratory-related death is elevated among those with evidence of overlapping disease compared to people with asthma alone; however, further prospective studies are needed to confirm the differential mortality burden of this population.

Overall an increased risk of CVD-related deaths was observed in patients with COPD (S4), however no significant excess incidence of CVD-related death at 10 years post diagnosis was observed when compared to people without COPD (Table 2). In our previous research, we observed a decline in the proportion of CVD deaths among patients with diagnosed COPD [12]. Among the UK general population, Bhatnager et al. report the age-standardised trends in CVD mortality, between 2005 and 2013 death rates declined in all UK regions from 400 per 100,000 population to 280 per 100,000 population [39]. The majority of the improvements were attributed to therapeutic and surgical interventions, and while the authors note that mortality has significantly improved, the prevalence of CVD has remained constant, evidenced by increases in rates of treatment and hospital admissions [39]. Further research adjusting for comorbid conditions, use of medication and hospital activity would be warranted to distinguish the influence of pre-existing CVD, and whether excess CVD-related mortality is driven by disease management or underlying disease mechanisms in patients with COPD.

### Strengths and limitations

One of the recommendations in the recent NHS Long Term plan [40] (in which chronic respiratory disease has been identified as a major area of focus for improvement) is the use of linked data sources to measure current standards of care [8] and aid direction of resources locally to reduce the COPD burden across the UK. As this is the first study to accurately estimate excess risk of respiratory-related death in a large sample of the English population using a competing risk approach, a major strength of this study is the use of linked primary care and morality data; in doing so we accurately compile mortality rates for people with a chronic disease, whereas, other common approaches rely on general population mortality statistics alone which underestimates chronic disease burden, as only around 40% of patients with chronic respiratory diseases have their disease recorded on their death certificates [12]. This approach could be adapted at local levels to evaluate the impact of interventions targeting reductions in respiratory mortality.

Linked electronic health record and mortality data has the advantage of providing further person-level information to control for confounding factors such as demographic data as well as area level deprivation which has been previously associated with wide variation in respiratory mortality [41]. CPRD is recognised as a reliable source for investigating UK general practice and health behaviours and has been used in over 1500 publications to date. Furthermore, mortality data are complete for all people and therefore we can be confident that mortality rate estimates are accurate [15, 42]. In addition, we included additional cohorts of overlap of asthma and COPD, for which there is limited mortality data [43], and expand on the findings reported in our previous study on mortality attributed to chronic respiratory disease [12].

However, our study has a number of limitations; it is possible that competing causes of death can be misrepresented in the mortality data since only a single underlying cause of death is identified on death certificates and the counterfactual situation (i.e. what someone would have died of if that particular cause had not been present) is unknown. Using the underlying cause of death omits the potential influence of secondary or tertiary causes; these may be informative if they fall within one of the major causes of interest (i.e., cardiovascular disease, any malignant neoplasm, diseases of the digestive system, mental and behavioural disease). Similarly, we do not include the receipt of treatments, which may explain the magnitude of the differences observed in the cumulative incidence at 10 years post diagnosis for non-respiratory-related causes of death between those with asthma or COPD and those without. Future research should utilise information in death certificates and health records to adjust for treatments and their contribution to risk of cause-specific death.

In addition, disease severity at diagnosis was not included in this analysis. The majority of people with asthma have mild disease and therefore we may underestimate excess mortality for only a small proportion of the asthma population, among whom mortality risk may be higher [44]. Clinical guidelines recommend that asthma is categorised based on medication usage and therefore future research should use an alternative study design to capture disease classification [3, 45]. A number of disease characteristics have been shown to be correlated with mortality risk among people with COPD. In the present study we did not include measures of disease severity however, the approach described in this research could be further expanded to stratify analysis according to frequency of exacerbations in the first year following diagnosis, post-bronchodilator lung function, as well as risk indices (e.g. BODE index [46]).

Our definition of ACO allows for people to have survived one disease in order to be diagnosed with the second, this may omit death that could have occurred between both diagnosis and potentially introduce bias; while this algorithm (S3) can reduce misclassification of overlap disease in electronic health records [7] we could not account for diagnostic uncertainty which may explain the variance in cause-specific mortality observed. We expect the impact of this to be minimal as the likelihood of observing a death within this diagnostic period would be low as patients will be more likely to be closely monitored following a new diagnosis of respiratory disease.

## Conclusions

This large cohort study demonstrates that physician diagnosed adult onset asthma and COPD are associated with an increased risk of respiratory death; however, there is a much greater 10-year probability of respiratory-related mortality among people with COPD compared to that for asthma after adjusting for competing risks of death from other causes. Further research including phenotypes of both asthma and COPD is warranted considering the clinical heterogeneity of ACO. These results support the argument that effective interventions are needed for people with a diagnosis of COPD in order to reduce their lifetime excess respiratory mortality risk.

## Supplementary Information

**Additional file 1**. Supplementary Figures and Tables.

## Data Availability

Data are available on request from the CPRD. Their provision requires the purchase of a license, and this license does not permit the authors to make them publicly available to all. This work used data from the version collected in September 2017 and have clearly specified the data selected each Methods section. To allow identical data to be obtained by others, via the purchase of a license, the code lists will be provided upon request. Licenses are available from the CPRD (http://www.cprd.com): The Clinical Practice Research Datalink Group, The Medicines and Healthcare products Regulatory Agency, 10 South Colonnade, Canary Wharf, London E14 4PU.

## Abbreviations

ACO: Asthma COPD overlap
BMI: Body mass index
COPD: Chronic obstructive pulmonary disease
CPRD: Clinical practice research datalink
CI: Confidence interval
CVD: Cardiovascular disease
HR: Subhazard ratio
ICD-10: International classification of disease version 10
IMD: Index of multiple deprivation
IQR: Inter-quartile range
NHS: National health service
ONS: Office for national statistics
UK: United kingdom
NRAD: National review of asthma deaths
