# Supplementary material for: Respiratory-related death in individuals with incident asthma and COPD: a competing risk analysis
Source: BMC Pulm Med. 2022 Jan 8;22:28. doi: 10.1186/s12890-022-01823-4 (PMC8742941; doi:10.1186/s12890-022-01823-4)
Supplement: Supplementary file 1 — Additional file 1. Supplementary Figures and Tables. [file 12890_2022_1823_MOESM1_ESM.docx]

# **Supplementary Material**

**Respiratory-related death in individuals with incident asthma and COPD: a competing risk analysis**

Alicia V Gayle, Cosetta Minelli, Jennifer K Quint

**Methods**

Validity of asthma and COPD diagnosis

This study relies on diagnostic codes only and not spirometry; this is based on two extensive validation studies (Quint et al, 2014, Nissen et al 2017) which demonstrated that the positive predicted values (PPV) of using diagnosis codes alone were sufficient at identifying disease (PPV 86.5% (95%CI 77.5–92.3%) for COPD, PPV 86.4% (95% CI 77.4% to 95.4%) for asthma).

The PPV for identifying diagnosed COPD and asthma were not greatly improved by using algorithms that are more complicated. For COPD diagnosis, requiring a diagnostic code, plus spirometry, plus specific COPD medication prescribed within 4 weeks of diagnosis; the PPV was slightly higher at 89.4% (80.7–94.5%) but reduced case numbers by 10%. For asthma, a combination of asthma code, medication prescription, evidence of reversibility testing did not result in a higher PPV (PPV 86.4%). Therefore, we believe that identification of disease was done to a reasonable standard.

References

Nissen F, Morales DR, Mullerova H, Smeeth L, Douglas IJ, Quint JK. Validation of asthma recording in the Clinical Practice Research Datalink (CPRD). BMJ open. 2017 Aug 1;7(8):e017474.

Quint JK, Müllerova H, DiSantostefano RL, Forbes H, Eaton S, Hurst JR, Davis K, Smeeth L. Validation of chronic obstructive pulmonary disease recording in the Clinical Practice Research Datalink (CPRD-GOLD). BMJ open. 2014 Jul 1;4(7).

**CPRD**

The CPRD provide primary care databases of de-identified medical records from general practitioners from 1987 to the present day. The CPRD primary care databases are rich sources of electronic health data for research, including data on demographics, symptoms, tests, diagnoses, therapies, health-related behaviours and referrals to secondary care. Data from practices that have consented to contribute data to CPRD are regularly uploaded to the CPRD servers. Data are then processed and go through quality checks before being pseudonymised and made available for research. Data are captured by GPs during routine clinical practice using the Vision or EMIS patient management software platform, clinical concepts are represented by ‘Read’ or ‘SNOMED CT’ codes (both are then mapped to unique ‘medcodes’), prescriptions are coded using the British National Formulary (BNF) and the Dictionary of Medicines and Devices (dm+d) codes, a subset of the SNOMED CT terminology, and have been assigned a “ProdCode.”, and in secondary care settings (including HES and ONS data linked to CPRD), ICD-10 codes are used.

**Data Linkage**

Data is linked by NHS Digital, the statutory trusted third party for linking data, using identifiable data held only by NHS Digital. Select GP practices consent to this process at a practice level, with individual patients having the right to opt-out. Use of HES and ONS data is Copyright © (2020), re-used with the permission of The Health & Social Care Information Centre, all rights reserved. This work is based in part on data from the Clinical Practice Research Datalink (CPRD) obtained under licence from the United Kingdom (UK) Medicines and Healthcare products Regulatory Agency (MHRA). The data is provided by patients and collected by the National Health Service (NHS) as part of their care and support. The interpretation and conclusions contained in this study are those of the authors alone.

**Table S1 COPD Codes**

|  | **CPRD Medcodes** |
| --- | --- |
| COPD codes additional to validated list | 104608, 104710, 104985, 105457, 106637, 65733, 67040, 60188, 46578, 16410 |

Validated list published as: Quint JK, Mullerova H, DiSantostefano RL, et al. Validation of chronic obstructive pulmonary disease recording in the Clinical Practice Research Datalink (CPRD-GOLD). *BMJ Open* 2014; **4**(7): e005540.

**Table S2 Asthma Codes**

| **CPRD Medcode** | **Term** |
| --- | --- |
| 78 | asthma |
| 81 | asthma monitoring |
| 185 | acute exacerbation of asthma |
| 232 | asthma attack |
| 233 | severe asthma attack |
| 1555 | bronchial asthma |
| 2290 | allergic asthma |
| 3018 | mild asthma |
| 3366 | severe asthma |
| 3458 | occasional asthma |
| 3665 | late onset asthma |
| 4442 | asthma unspecified |
| 4606 | exercise induced asthma |
| 4892 | status asthmaticus nos |
| 5267 | intrinsic asthma |
| 5627 | hay fever with asthma |
| 5798 | chronic asthmatic bronchitis |
| 5867 | exercise induced asthma |
| 6707 | extrinsic asthma with asthma attack |
| 7058 | emergency admission, asthma |
| 7146 | extrinsic (atopic) asthma |
| 7191 | asthma limiting activities |
| 7378 | asthma management plan given |
| 7416 | asthma disturbing sleep |
| 7731 | pollen asthma |
| 8335 | asthma attack nos |
| 8355 | asthma monitored |
| 9018 | number of asthma exacerbations in past year |
| 9552 | change in asthma management plan |
| 9663 | step up change in asthma management plan |
| 10043 | asthma annual review |
| 10274 | asthma medication review |
| 10487 | asthma - currently active |
| 11370 | asthma confirmed |
| 12987 | late-onset asthma |
| 13064 | asthma severity |
| 13065 | moderate asthma |
| 13175 | asthma disturbs sleep frequently |
| 13176 | asthma follow-up |
| 14777 | extrinsic asthma without status asthmaticus |
| 15248 | hay fever with asthma |
| 16070 | asthma nos |
| 16667 | asthma control step 2 |
| 16785 | asthma control step 1 |
| 18223 | step down change in asthma management plan |
| 18224 | asthma control step 3 |
| 18323 | intrinsic asthma with asthma attack |
| 19167 | asthma monitoring by nurse |
| 19519 | asthma treatment compliance unsatisfactory |
| 19520 | asthma treatment compliance satisfactory |
| 19539 | asthma monitoring check done |
| 20860 | asthma control step 5 |
| 20886 | asthma control step 4 |
| 21232 | allergic asthma nec |
| 22752 | occupational asthma |
| 24479 | emergency asthma admission since last appointment |
| 24506 | further asthma - drug prevent. |
| 24884 | asthma causes daytime symptoms 1 to 2 times per week |
| 25181 | asthma restricts exercise |
| 25791 | asthma clinical management plan |
| 26501 | asthma never causes daytime symptoms |
| 26503 | asthma causes daytime symptoms most days |
| 26504 | asthma never restricts exercise |
| 26506 | asthma severely restricts exercise |
| 26861 | asthma sometimes restricts exercise |
| 27926 | extrinsic asthma with status asthmaticus |
| 29325 | intrinsic asthma without status asthmaticus |
| 30458 | asthma monitoring by doctor |
| 30815 | asthma causing night waking |
| 31167 | asthma night-time symptoms |
| 31225 | asthma causes daytime symptoms 1 to 2 times per month |
| 38143 | asthma never disturbs sleep |
| 38144 | asthma limits walking up hills or stairs |
| 38145 | asthma limits walking on the flat |
| 38146 | asthma disturbs sleep weekly |
| 39478 | wood asthma |
| 39570 | asthma causes night symptoms 1 to 2 times per month |
| 40823 | brittle asthma |
| 41017 | aspirin induced asthma |
| 41020 | absent from work or school due to asthma |
| 42824 | asthma daytime symptoms |
| 45073 | intrinsic asthma nos |
| 45782 | extrinsic asthma nos |
| 46529 | attends asthma monitoring |
| 47337 | asthma accident and emergency attendance since last visit |
| 47684 | detergent asthma |
| 58196 | intrinsic asthma with status asthmaticus |
| 73522 | work aggravated asthma |
| 93353 | sequoiosis (red-cedar asthma) |
| 93736 | royal college of physicians asthma assessment |
| 98185 | asthma control test |
| 99793 | patient has a written asthma personal action plan |
| 100107 | health education - asthma self management |
| 100397 | asthma control questionnaire |
| 100509 | under care of asthma specialist nurse |
| 100740 | health education - structured asthma discussion |
| 102170 | asthma review using roy colleg of physicians three questions |
| 102209 | mini asthma quality of life questionnaire |
| 102301 | asthma trigger - seasonal |
| 102341 | asthma trigger - pollen |
| 102395 | asthma causes symptoms most nights |
| 102400 | asthma causes night time symptoms 1 to 2 times per week |
| 102449 | asthma trigger - respiratory infection |
| 102713 | asthma limits activities 1 to 2 times per month |
| 102871 | asthma trigger - exercise |
| 102888 | asthma limits activities 1 to 2 times per week |
| 102952 | asthma trigger - warm air |
| 103318 | health education - structured patient focused asthma discuss |
| 103321 | asthma trigger - animals |
| 103612 | asthma never causes night symptoms |
| 103631 | royal college physician asthma assessment 3 question score |
| 103813 | asthma trigger - cold air |
| 103944 | asthma trigger - airborne dust |
| 103945 | asthma trigger - damp |
| 103952 | asthma trigger - emotion |
| 103955 | asthma trigger - tobacco smoke |
| 103998 | asthma limits activities most days |
| 105420 | asthma self-management plan review |
| 105674 | asthma self-management plan agreed |
| 106805 | chronic asthma with fixed airflow obstruction |
| 107167 | number days absent from school due to asthma in past 6 month |

**Figure S3: Study Design**

**
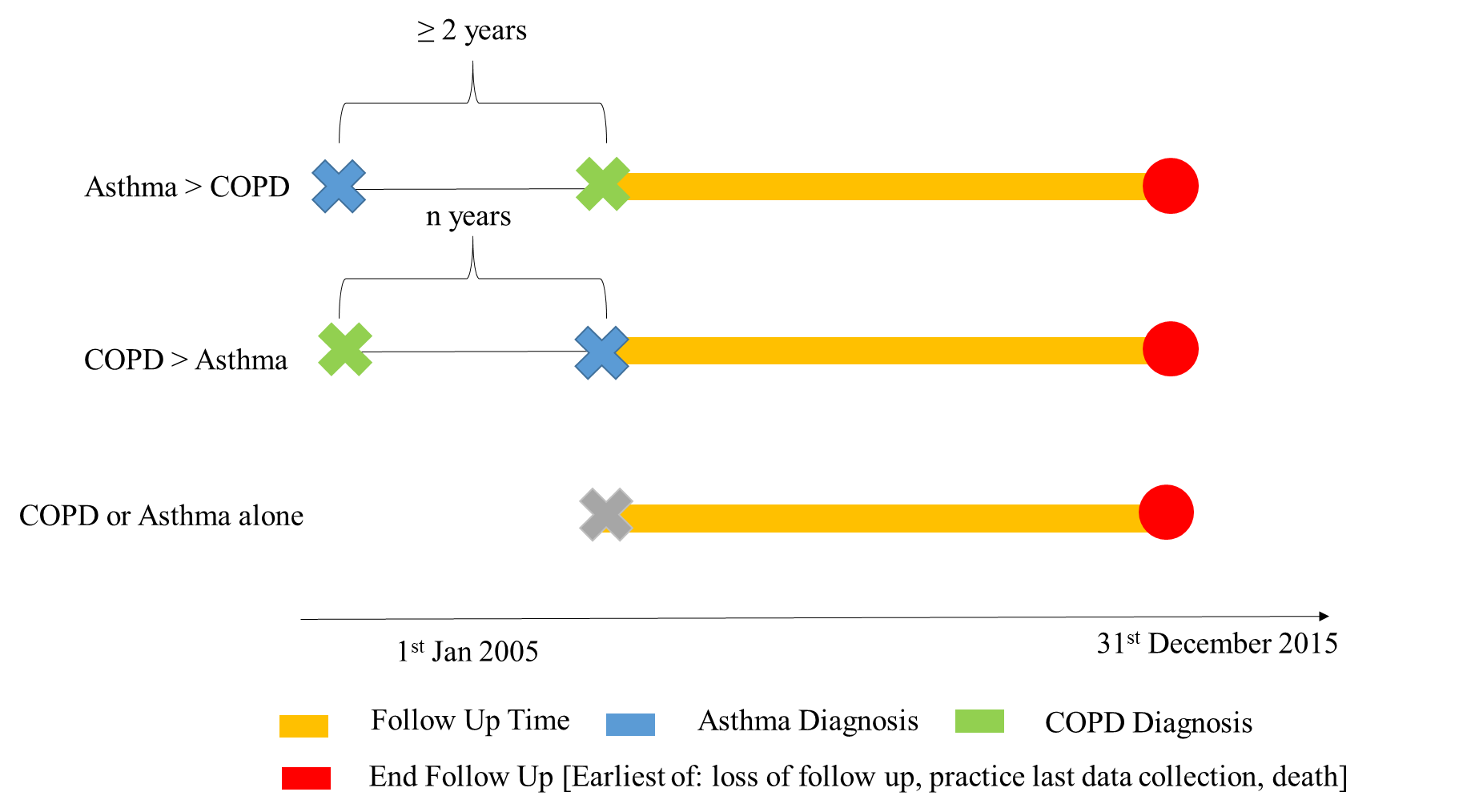
**

Validation of ACO definition published as: Nissen F, Morales DR, Mullerova H, Smeeth L, Douglas IJ, Quint JK. Concomitant diagnosis of asthma and COPD: a quantitative study in UK primary care. Br J Gen Pract. 2018;68(676):e775-e82.

**Table S4 Mortality rates per 100,000 person-years by disease and ICD-10 cause of death**

|  | | | **Disease** | | **The general population** | |  | |  |  |  |
| --- | --- | --- | --- | --- | --- | --- | --- | --- | --- | --- | --- |
| **Disease** | **Cause of death** | | **deaths** | **rate** | **deaths** | **rate** | | **Hazard Ratio (95%CI)** | | **p-value** | |
| **Asthma** | | | n = 65,021 | | n = 324,885 | |  | |  |  |  |
|  | All-cause mortality | | 7,671 | 2,954 | 29,703 | 2,034 | | ~ | |  | |
|  | Diseases of the respiratory system (J00-J99) | | 1,867 | 719 | 3,549 | 243 | | 2.22 (2.08 - 2.36) | | <0.0001 | |
|  | Diseases of the circulatory system (I00-I99) | | 2,051 | 790 | 8,678 | 594 | | 0.98 (0.93 - 1.03) | | 0.479 | |
|  | Neoplasms (C00-D48) | | 2,234 | 860 | 9,096 | 623 | | 1.10 (1.04 - 1.16) | | <0.0001 | |
|  | Diseases of the digestive system (K00-K93) | | 389 | 150 | 1,456 | 100 | | 1.13 (1.00 - 1.28) | | 0.056 | |
|  | Mental and behavioural disorders (F00-F99) | | 316 | 122 | 2,660 | 182 | | 0.55 (0.49 - 0.63) | | <0.0001 | |
|  | Other* | | 814 | 313 | 4,264 | 292 | | 0.82 (0.76 - 0.89) | | <0.0001 | |
| **COPD** | | | n = 45,649 | | n = 228,076 | |  | |  |  |  |
|  | All-cause mortality | | 15,800 | 8,173 | 46,036 | 4,631 | | ~ | |  | |
|  | Diseases of the respiratory system (J00-J99) | | 4,656 | 2,409 | 5,058 | 509 | | 3.31 (3.15 - 3.47) | | <0.0001 | |
|  | Diseases of the circulatory system (I00-I99) | | 4,075 | 2,108 | 14,398 | 1,448 | | 1.00 (0.96 - 1.04) | | 0.911 | |
|  | Neoplasms (C00-D48) | | 4,606 | 2,383 | 13,536 | 1,362 | | 1.16 (1.11 - 1.20) | | <0.0001 | |
|  | Diseases of the digestive system (K00-K93) | | 631 | 326 | 2,070 | 208 | | 0.98 (0.88 - 1.08) | | 0.663 | |
|  | Mental and behavioural disorders (F00-F99) | | 487 | 252 | 4,551 | 458 | | 0.42 (0.38 - 0.47) | | <0.0001 | |
|  | Other | | 1,345 | 696 | 6,423 | 646 | | 0.79 (0.74 - 0.84) | | <0.0001 | |
| CI: confidence interval. * Includes missing cause of death. Subdistribution hazard ratios were adjusted for Age, gender, smoking, BMI, IMD, CCI and other causes of death as competing risks. Standard errors estimated using robust method | | | | | | | | | | | |
|  | |  |  |  |  |  |  |  |  |  |  |

**Table S5 Most common 15 respiratory-related causes of death by disease**

| **Cause of Death (ICD-10 J00 – J99)** | **Asthma** | | **COPD** | | **ACO** |
| --- | --- | --- | --- | --- | --- |
|  | **Yes** | **No** | **Yes** | **No** |  |
|  | (N=1,867) | (N=3,549) | (N=4,656) | (N=5,058) | (N=1,429) |
| Chronic obstructive pulmonary disease, unspecified (J44.9) | 31.0% | 12.6% | 33.6% | 5.3% | 32.9% |
| Chronic obstructive pulmonary disease with acute lower respiratory infection (J44.0) | 22.4% | 11.1% | 29.0% | 6.5% | 28.1% |
| Pneumonia, unspecified (J18.9) | 8.6% | 22.5% | 5.8% | 27.5% | 6.3% |
| Bronchopneumonia, unspecified (J18.0) | 6.9% | 20.0% | 5.0% | 23.1% | 5.4% |
| Chronic obstructive pulmonary disease with acute exacerbation, unspecified (J44.1) | 6.5% | 2.4% | 7.6% | 1.3% | 8.0% |
| Other interstitial pulmonary diseases with fibrosis (J84.1) | 5.7% | 6.4% | 5.2% | 8.6% | 2.9% |
| Bronchiectasis (J47) | 3.3% | 2.1% | 2.1% | 1.9% | 4.2% |
| Asthma, unspecified (J45.9) | 3.2% | 0.5% | 0.4% | 1.2% | 2.8% |
| Emphysema, unspecified (J43.9) | 2.4% | 1.4% | 3.5% | 0.8% | 2.1% |
| Unspecified acute lower respiratory infection (J22) | 1.8% | 4.8% | 0.8% | 5.5% | 1.2% |
| Pneumonitis due to food and vomit (J69.0) | 1.7% | 4.3% | 1.2% | 4.5% | 0.9% |
| Other specified respiratory disorders (J98.8) | 1.6% | 5.1% | 1.4% | 5.6% | 1.5% |
| Lobar pneumonia, unspecified (J18.1) | 1.4% | 1.9% | 1.1% | 2.7% | 0.3% |
| Interstitial pulmonary disease, unspecified (J84.9) | 1.2% | 1.4% | 0.8% | 1.6% | 0.6% |
| Other specified chronic obstructive pulmonary disease (J44.8) | 0.6% | 0.2% | 0.6% | 0.1% | 0.5% |

**Table S6 Mortality rates per 100,000 person-years by disease and ICD-10 cause of death among those with overlap of asthma and COPD**

|  | ACO  (N = 22,145) | |
| --- | --- | --- |
|  | deaths | rate |
| All-cause mortality | 4,307 | 4,485 |
| Diseases of the respiratory system (J00-J99) | 1,429 | 1,488 |
| Diseases of the circulatory system (I00-I99) | 1,081 | 1,126 |
| Neoplasms (C00-D48) | 1,075 | 1,119 |
| Diseases of the digestive system (K00-K93) | 212 | 221 |
| Mental and behavioural disorders (F00-F99) | 142 | 148 |
| Other | 368 | 383 |

**Figure S7 Subhazard of respiratory-related mortality between disease groups by order of overlapping diagnosis**


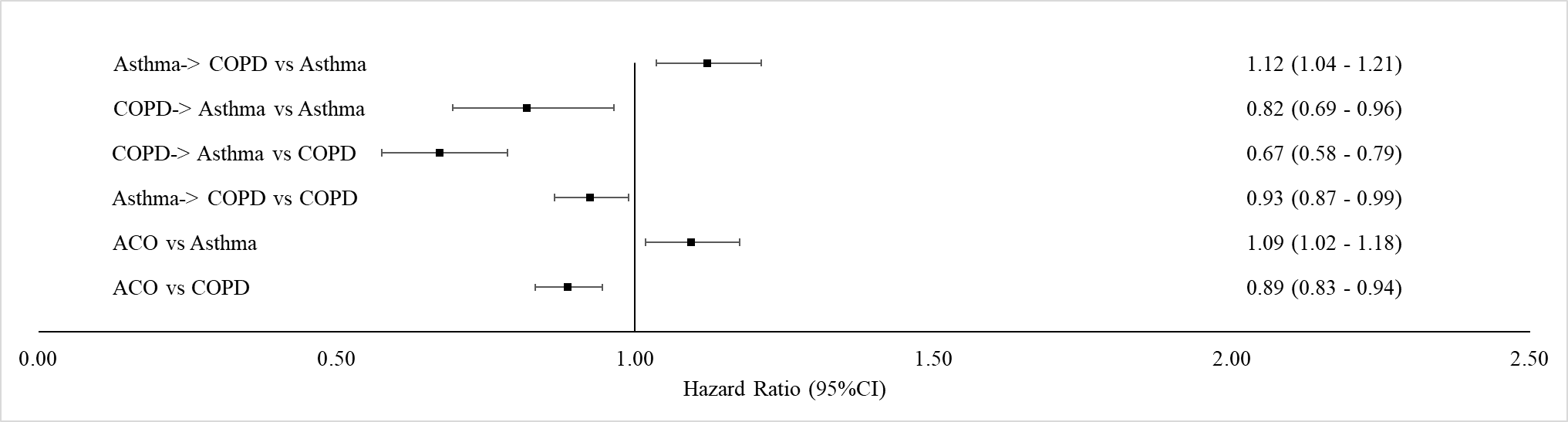


- Denotes order of diagnosis (see Figure S3 for definitions), ACO: Asthma COPD overlap, CI: Confidence Interval
